# Bacillus Calmette-Guérin vaccine-induced lupus vulgaris in a 3-year-old child^☆^^☆☆^

**DOI:** 10.1016/j.abd.2020.07.022

**Published:** 2021-07-14

**Authors:** Nisha V. Parmar, Amani Al Falasi, Alia Almualla

**Affiliations:** Dermatology Centre, Dubai Health Authority, Dubai, United Arab Emirates

Dear Editor,

A 3-year-old girl presented with an asymptomatic lesion on the left upper arm of 2 years’ duration. The lesion started as a papule at the site of Bacillus Calmette-Guérin (BCG) vaccination scar at the age of 1 year and gradually enlarged to its present size. She had no constitutional symptoms and was otherwise well. There was no history of contact with an active case of Tuberculosis (TB). General and systemic examinations were normal. Cutaneous examination revealed an erythematous plaque measuring 5×4 cm with thick adherent scales and fingerlike extensions on the left deltoid region (Fig. 1). Clinical differential diagnoses considered were BCG vaccine-induced lupus vulgaris, chromoblastomycosis, psoriasis, and discoid lupus erythematosus.

**Figure 1:**
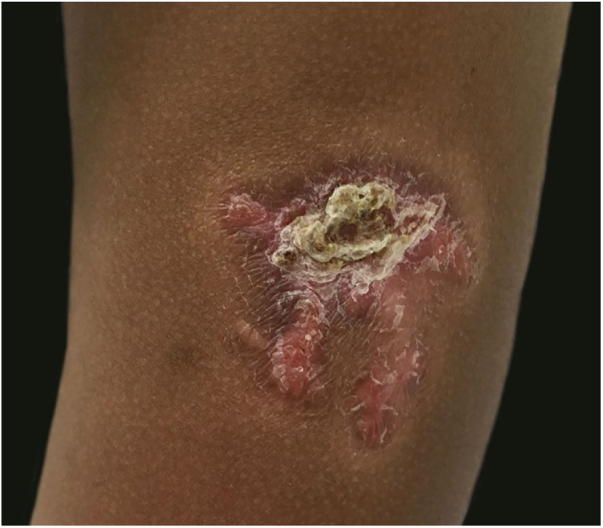
Well-demarcated erythematous scaly plaque with centrally adherent thick scales and finger-like extensions measuring 5×4 cm at site of BCG vaccine scar on left deltoid region.

Routine hematological investigations showed a raised erythrocyte sedimentation rate. Her chest X-Ray was normal. Serology for HIV was negative. A skin biopsy of the plaque revealed non-caseating epithelioid cell granulomas with a rim of lymphocytes in the upper and mid dermis (Fig. 2). Acid-fast bacilli and periodic acid-Schiff staining of biopsy specimen were negative. Cultures of the skin biopsy were also negative for mycobacteria and fungi. Mycobacterium (M) tuberculosis complex DNA detection via Polymerase Chain Reaction (PCR) of the biopsy specimen was negative. Interferon-gamma release assay using the Quantiferon TB gold test was also negative. Based on the site of lesion, clinical morphology, and presence of epithelioid cell granulomas in histopathology, the possibility of BCG vaccine-induced lupus vulgaris was considered and a trial of antitubercular therapy was commenced. *M. bovis* is inherently resistant to pyrazinamide and ethambutol is avoided in children due to its ophthalmologic side effects.^1^ The child thus received a regimen consisting of rifampicin (6 mg/kg/day) and isoniazid (5 mg/kg/day) from the Pediatric Infectious Disease unit. The lesion healed completely after 2 months of therapy (Fig. 3). A final diagnosis of BCG vaccine-induced lupus vulgaris was thus made.

**Figure 2:**
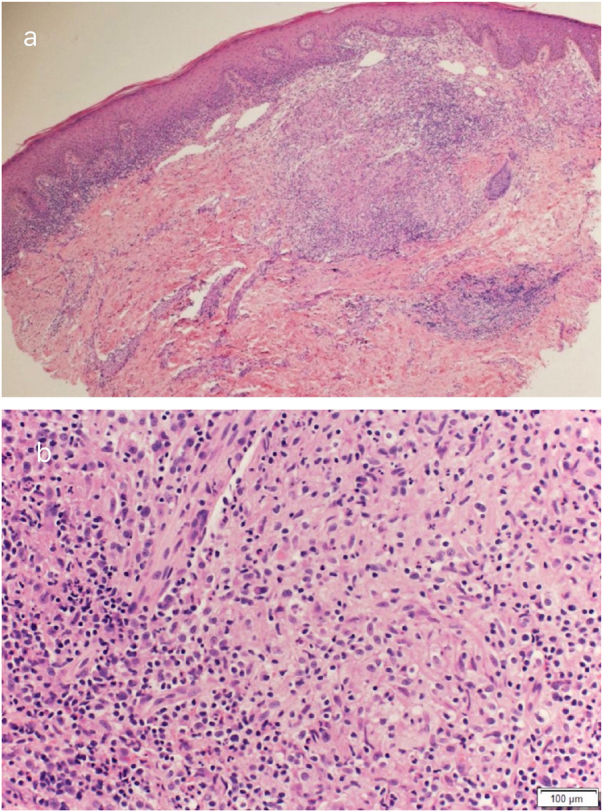
(A), Granulomatous infiltration in upper and mid-dermis. (B), Non-caseating epithelioid cell granuloma with peripheral lymphocytic rim.

**Figure 3:**
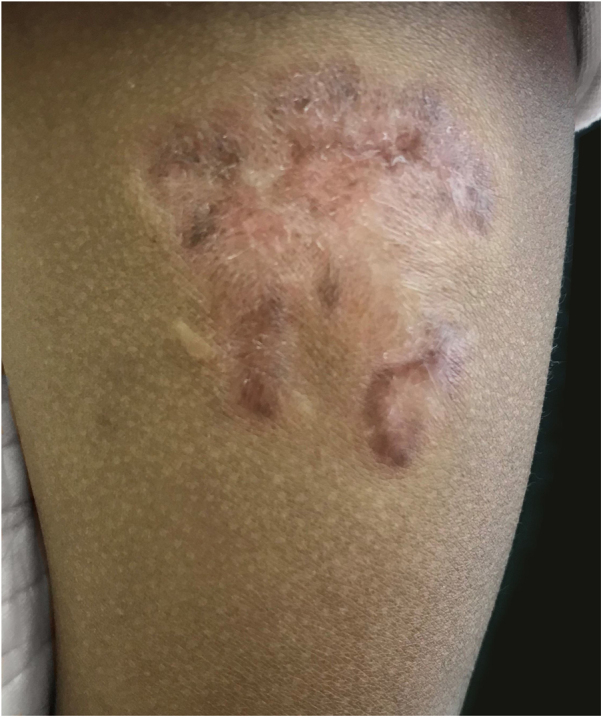
Scar at site of lesion post-treatment.

The BCG vaccine is composed of a live attenuated strain of *M. bovis*. It is routinely administered to all neonates in high endemic countries to prevent the more serious forms of tubercular infections such as meningoencephalitis and miliary tuberculosis in children.

Cutaneous complications of the BCG vaccine in the immunocompetent host include the common local reaction to the vaccine and uncommon reactions. The local reaction occurs 2–6 weeks post-vaccination as a small papule that may discharge purulent material, enlarge to form a shallow ulcer which scars. The rare complications include BCG granuloma, injection site keloids, lupus vulgaris, scrofuloderma, tuberculids, fixed drug eruption, and BCG granulomas occurring during the course of Kawasaki disease.^2^

BCG vaccine-induced lupus vulgaris occurs in 5 per million vaccinations.^3^ It is commoner after multiple vaccinations and usually manifests after 1 year of vaccination. Lupus vulgaris is a paucibacillary form of cutaneous tuberculosis that occurs either via hematogenous spread or direct inoculation. A skin biopsy is helpful in obtaining a histopathological diagnosis. Other confirmatory tests for tuberculosis may be inconclusive as in our patient. A high index of suspicion is required for the diagnosis and a trial of antitubercular drugs is warranted.

## Financial support

None declared.

## Authors’ contributions

Nisha V Parmar: Conception and planning of the study; critical review of the literature; obtaining, analysis, and interpretation of the data; elaboration and writing of the manuscript; critical review of the manuscript. approval of the final version of the manuscript.

Amani AlFalasi: Approval of the final version of the manuscript; obtaining, analysis, and interpretation of the data; critical review of the manuscript.

Alia AlMualla: Approval of the final version of the manuscript; obtaining, analysis, and interpretation of the data; critical review of the manuscript.

## Conflicts of interest

None declared.
